# Evaluating Autoxidation Radical Scavengers and Additives to Enhance Aminopolymer Sorbent Stability

**DOI:** 10.1021/acs.energyfuels.5c04042

**Published:** 2025-12-02

**Authors:** Yoseph A. Guta, Paco Tang, Sichi Li, Jiaqi Zhang, Miles A. Sakwa-Novak, Simon H. Pang, Carsten Sievers, Christopher W. Jones

**Affiliations:** † School of Chemical and Biomolecular Engineering, 1372 Georgia Institute of Technology, Atlanta, Georgia 30332, United States; ‡ Materials Science Division, 4578 Lawrence Livermore National Laboratory, Livermore, California 94550, United States; § Virdis Systems, Inc., 2342 Broadway, San Francisco, California 94115, United States

## Abstract

Solid amine sorbents have shown promise in the removal of ultradilute CO_2_ from the atmosphere. Despite being a promising candidate material type for this application, these sorbents are prone to degradation during long-term exposure to environmental components such as CO_2_, O_2_, and H_2_O, with amine oxidation being a particularly challenging problem. In this study, we investigate the potency of different radical scavengers and additives in mitigating the degradation of a model poly­(ethylenimine) (PEI)/Al_2_O_3_ sorbent under direct air capture (DAC)-relevant conditions. The results reveal that a 4,4′-bis­(α,α-dimethylbenzyl)­diphenylamine (BDDPA)-incorporated PEI/Al_2_O_3_ sorbent showed the most resistance toward oxidative degradation at varying exposure times and BDDPA loadings under CO_2_-free air (21% O_2_/balance N_2_) at 120 °C. Interestingly, under humid (∼43% relative humidity (RH) at 26 °C) and dry 0.04% CO_2_-air, the BDDPA/PEI/Al_2_O_3_ sorbent showed enhanced sorbent stability both at 70 and 120 °C after 4.5 h of exposure. Under humid CO_2_-free air, at 120 °C, the antioxidant performance slightly declined (in comparison to the dry CO_2_-free air condition) but displayed a much higher stability than the pristine sorbent. Overall, the ability of BDDPA to inhibit sorbent degradation under dry and humid, CO_2_-free and CO_2_-containing (0.04%) air at intermediate (70 °C) and elevated (120 °C) temperatures is promising in prolonging sorbent stability and underscores the importance of performing accelerated oxidation studies in the presence of all species that are expected to be present in DAC processes to identify suitable stabilization treatments for sorbent materials.

## Graphical abstract

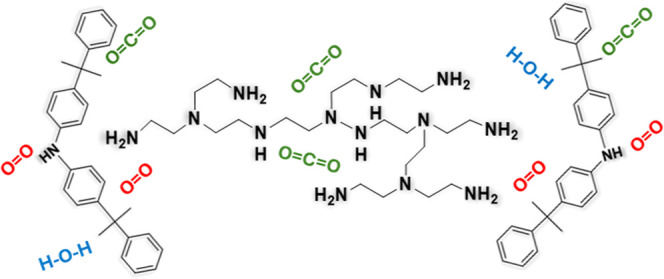

## Introduction

Greenhouse gas (GHG) emissions are the primary cause of global climate change, with the major impact stemming from the emissions of CO_2_ resulting from the use of fossil fuels.^1,2^ Global CO_2_ concentrations have increased by 150% since the Industrial Revolution began in 1750.^3,4^ Despite global efforts to reduce carbon emissions through increased use of renewable energy and technological innovations, the carbon budget needed to limit global temperature rise to 1.5 °C is projected to be exceeded within the next seven years.^5^ With the need to rapidly reduce carbon emissions globally, new technologies must be developed that are versatile and resilient to combat the contemporary effects of climate change.^6^

One of the most promising technologies to reduce carbon emissions from the atmosphere is direct air capture (DAC). DAC technologies can be widely used in various settings and locations due to their spatial flexibility and adaptability and their ability to be combined with low-carbon energy sources such as geothermal energy.^7^ Hence, the development and utilization of DAC for CO_2_ capture will be an essential step in mitigating the effects of climate change, with the potential to achieve carbon negativity.^8,9^ Although DAC comes with its own set of challenges, such as the energy needed for operation and the logistics of its use and carbon storage, the right combination of materials and implementation may allow for the mitigation of carbon emissions and climate change.^10^

While there are a wide range of sorbents that can be used for DAC applications, solid-amine-functionalized sorbents have been most extensively studied due to their high CO_2_ selectivity and capacity, relatively low energy requirements during use, and controllable physical properties of the solid sorbent support.^11,12^ One of the main hurdles for aminopolymer sorbents in large-scale deployment and commercialization is their tendency to lose their CO_2_ capacity after multiple adsorption–desorption cycles due to oxidation associated with the high atmospheric oxygen concentration (∼21%) and the elevated temperatures during sorbent regeneration (80–120 °C).^13–16^ Recent studies have shown that other atmospheric components such as CO_2_ and H_2_O vapor can have complex contributions to sorbent stability or degradation, which can lead to accelerated or slowed oxidative degradation.^17–19^

To enable the long-term stability of aminopolymer sorbents, studies have explored the implementation of a wide range of mitigation techniques. Some of the main mitigation techniques involve designing/developing new aminopolymers/amine species resistant to oxidative degradation,^15,16,20–23^ incorporating additives (phosphate salts,^24^ ether compounds,^25^ common and sulfur-containing antioxidants^26^) with aminopolymer sorbents and modification or functionalization of the aminopolymers.^14,27–35^

The incorporation of compounds with hydroxyl^25,26,28,30,36,37^ or ether^25^ groups and the functionalization of aminopolymers with epoxides^31,32,34,35,38^ or ethers^31^ is reported to enhance oxidative stability by forming hydrogen bonds with the amine sites of the aminopolymer. The hydrogen bonds limit the interactions with free radical species that can promote oxidation, while restricting the movement of the amine chains (reducing mobility), slowing down radical propagation and limiting O_2_ diffusion. PEG has been reported to enhance the CO_2_ adsorption capacity by PEI aggregate intercalation and displacement of PEI–wall interactions, allowing for additional amines to be available for CO_2_ adsorption.^39^ Vu et al. showed sulfur-containing additives reduce the oxidative degradation of PEI/SiO_2_ sorbents by decomposing active hydroperoxide species produced during the oxidation to alcohols.^26^ Similarly, Wang et al. showed that 2-mercaptobenzimidazole antioxidants inhibit oxidative degradation of a TEPA-MCM-41 sorbent by scavenging free radicals and hydroperoxides and forming stable alcohols.^27^ Another approach reported by Choi et al.^14^ uses chelating agents to trap parts per million-level metal impurities together with epoxy-modified PEI to enhance oxidation stability in O_2_-containing flue gas. Similar work from the same group also showed that enhanced sorbent stability can be achieved by incorporating monocationic phosphate salts to inhibit the activity of metal impurities during oxidation.^24^

Advances in these approaches may allow for prolonging the sorbent lifetime, facilitating the large-scale deployment of amine-based sorbents with lower costs. However, many of the experimental conditions explored in these studies, which often use accelerated oxidation testing with simple gas compositions in the absence of CO_2_ or H_2_O, do not directly translate to DAC operational conditions. As we develop more understanding of the degradation mechanism(s) of aminopolymer sorbents and the roles CO_2_, H_2_O, and other environmental components play in accelerating the degradation,^17–19^ identifying/exploring new mitigation techniques or testing already existing additives, antioxidants, or other techniques in the presence of these key environmental components is necessary.

In this study, we investigate the effectiveness of radical scavengers and other stabilizing additives commonly used to mitigate light, heat, or impurity-induced degradation in commercial hydrocarbon polymers for their ability to inhibit aminopolymer-sorbent degradation under temperatures and gas mixtures used in DAC processes. These molecules were selected because they specifically target radical species that form during the degradation of aminopolymer sorbents, such as alkyl-peroxyl radicals. The stability of additive-incorporated PEI/Al_2_O_3_ sorbents under dry and humid CO_2_-free air (21% O_2_ balance N_2_) and CO_2_ (400 ppm)-containing air is explored using thermogravimetric analysis (TGA) and in situ DRIFTS-IR spectroscopy techniques. The findings from this work identify radical scavengers/additives that are promising in enhancing the long-term stability of amine-based sorbents and highlight the importance of investigating sorbent stability under temperatures and gas mixtures used in DAC processes in the development of mitigation techniques for sorbent degradation.

## Experimental Section

### Material Preparation

The additives explored for sorbent stability enhancement were 4,4′-bis­(α,α-dimethylbenzyl)­diphenylamine (BDDPA, 98% Sigma-Aldrich), bis­(4-*tert*-butylphenyl)­amine (BTBPA, >90% TCI America), bis­(2,2,6,6-tetra-methyl-4-piperidinyl) sebacate (BTMPS, >98% Sigma-Aldrich), and 2,4,6-tri*tert*-butylnitroso-benzene (TTBNB, >95% Fisher Scientific) (Figure 1) and were used as purchased. For the additive-sorbent composite synthesis, the desired amount of PEI 800 g/mol branched poly­(ethylenimine) (PEI, Sigma-Aldrich) and the desired amount of the respective additive (BTBPA, BTMPS, BDDPA, or TTBNB) were separately dissolved in methanol in 20 mL vials on a stirring plate. After being fully dissolved, the PEI/methanol and the additive/methanol solutions were added into a round-bottomed flask and mixed for about 30 min on a stirring plate. Following the mixing, the PEI/additive/methanol solution was added to a round-bottomed flask containing the desired amount of mesoporous γ-Al_2_O_3_ (CATALOX HP 14/150 alumina, Sasol) support. The γ-Al_2_O_3_ was dried in an oven at 120 °C overnight. The slurry mixture was stirred overnight on a stirring plate set at 400 rpm at room temperature. After the overnight stirring, methanol was removed by rotary evaporation, and the remaining additive-sorbent composite powder was dried overnight under vacuum (∼20 mTorr). The ^13^C CPMAS NMR spectra in Figure S1 show incorporation of the additives into the sorbent.

**Figure 1:**
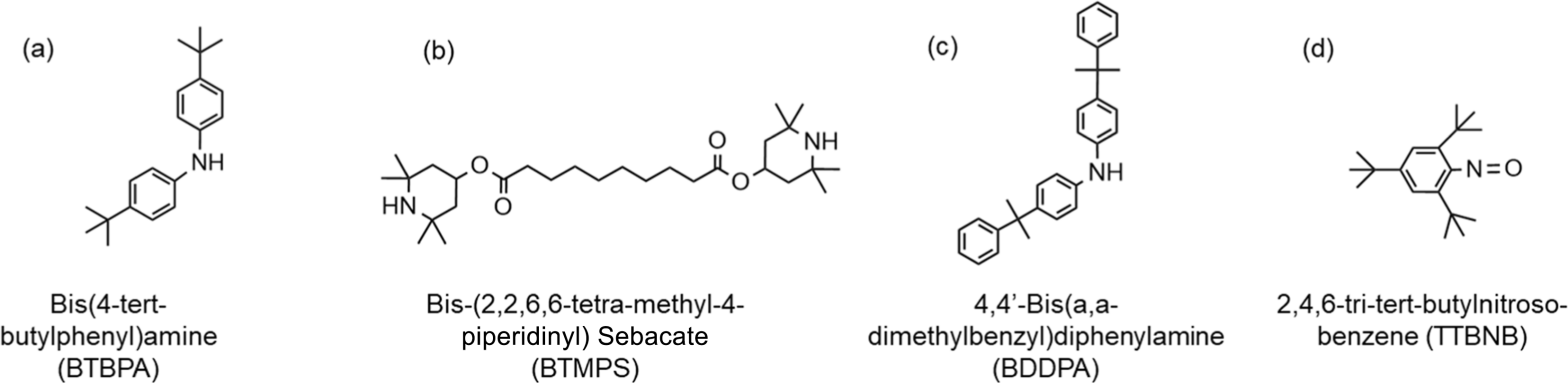
Chemical structures for radical scavengers used in this study.

Each sorbent incorporates 2 mol % additive molecule, except for the pristine sorbent. In terms of mass fraction, each sorbent contained 35 wt % PEI and 5 wt % BDDPA, 4 wt % BTBPA, 6 wt % BTMPS, or 4 wt % TTBNB. The pristine sorbent contained 35 wt % PEI. All gas mixtures used in this study were purchased from Airgas.

### Sorbent Characterization

The total organic content of the sorbents was determined using thermogravimetric combustion analysis (TGA 550, TA Instruments), while the pore filling was calculated based on N_2_ physisorption isotherm measurements. The N_2_ physisorption isotherm experiments were conducted using a Micromeritics TriStar II 3020 Version 3.02 at 77 K. Before the analysis, all samples were pretreated at 60 °C under vacuum for more than 10 h to remove weakly adsorbed species. The amounts removed are equivalent to the mass loss observed during the TGA pretreatment procedure for CO_2_ adsorption or sorbent deactivation experiments (1 h under N_2_ and at 100 °C). The BET surface area and the BHJ adsorption pore volume of the support and the sorbents are listed in Table S1.

For the organic combustion experiments, each sorbent was pretreated by heating to 100 °C for 1 h under N_2_ to remove weakly adsorbed species. After the pretreatment step, the combustion experiment was conducted by heating the sample to 900 °C at 10 °C/min under air (Airgas, Ultra-Zero Air, 21% O_2_ balance N_2_). The mass loss during the combustion process was considered as the organic content of the sorbent containing PEI for the pristine sorbent and PEI and additives for the sorbents incorporating one of the four modifiers. Table S2 shows the expected and measured organic loadings.

The ^13^C CPMAS NMR spectra were collected using a Bruker AVANCE III 400 MHz spectrometer with a 9.4 T wide-bore magnet. Peaks were referenced with respect to adamantane (38.45 ppm). A total 8192 scans were recorded at a magic angle spinning (MAS) rate of 12 kHz. In all experiments, around 90 mg of powder samples were packed into a 4 mm zirconia rotor.

### Sorbent Deactivation Experiments

Sorbent deactivation experiments were performed using a TGA 550 apparatus (TA Instruments). Before the sorbent deactivation step, about 29 ± 0.05 mg of the respective sorbent was loaded into a 50 μL high-temperature platinum pan and pretreated under N_2_ (100 mL/min) at 100 °C for 60 min. After the pretreatment, the sorbent was heated at 5 °C/min to the desired set point temperature under N_2_ (100 mL/min) and exposed to dry or humid CO_2_-free air (21% O_2_/balance N_2_) or 0.04% CO_2_-air at 70 or 120 °C for the target exposure period. For humid experiments, the gas mixtures (CO_2_-free air or 0.04% CO_2_-air) were prehumidified by flowing through bubblers containing saturated K_2_CO_3_ solution at 26 °C to achieve ∼43% relative humidity. Following the deactivation step, the sorbent was cooled to 25 °C at 20 °C/min under N_2_ (100 mL/min).

### CO_2_ Adsorption Experiments

The CO_2_ adsorption capacity of the fresh and degraded sorbents was measured by using a TGA (TA Instruments Q500) instrument. The change in the CO_2_ adsorption capacity before and after exposure to a deactivating environment was used to determine the sorbent deactivation.

Before the CO_2_ adsorption experiments, about ∼20 mg of sorbent was loaded onto a 50 μL platinum pan and pretreated under N_2_ gas (90 mL/min) (Airgas UHP300) at 100 °C for 60 min. Weakly sorbed species were removed during the pretreatment step. After the pretreatment, the sorbent was cooled to 30 °C at 10 °C/min under a He or N_2_ gas. For the CO_2_ adsorption measurements, the sorbent was exposed to 400 ppm of CO_2_ (balance He or N_2_) at 90 mL/min for 3 h. The mass gain during the 3 h of adsorption was attributed to the adsorption of CO_2_ by the sorbent. The adsorbed CO_2_ was removed by heating the sorbent isothermally under He or N_2_ gas (90 mL/min) at 100 °C for 1 h. Following the desorption step, the sorbent was cooled to 30 °C.

### In Situ FTIR Spectroscopy

In situ DRIFTS FTIR spectroscopy experiments were conducted by using a Thermo Scientific Nicolet iS10 FTIR spectrometer. Changes in the molecular structure of the sorbent were tracked to observe the impact of different gas mixtures at varying temperatures.

Before every experiment, ∼40 mg of sorbent was loaded into the DRIFTS cell and purged for ∼20 min under N_2_ (80 mL/min) at room temperature (∼21 °C). Following N_2_ purging, the sorbent was heated to 100 °C and held at 100 °C for 1 h under N_2_ at 100 mL/min to remove weakly sorbed species. Following sorbent activation, the temperature was set to the desired temperature under a N_2_ flow at 80 mL/min. When the target temperature was reached and stabilized, the flow was switched to the desired gas mixture, CO_2_-free air (21% O_2_/balance N_2_) or CO_2_-air (0.04% CO_2_ balance air) mixtures at 80 mL/min. The sorbent was held at the target temperature for a desired time. During the exposure to the gas mixtures, sample spectra were collected for the entire experimental period by using Thermo Scientific Omnic software. The IR spectra illustrated in this study represent changes that occurred due to the exposure, as spectral contributions from the sample prior to exposure are subtracted from the spectra after the exposure.

### Density Functional Theory Calculations

Initial molecular structures of triethylenetetramine (TETA) and BDDPA were generated using the Gen3D function for conformer searching in the Open Babel package.^40^ To prepare for bond dissociation energy (BDE) calculations, hydrogen atoms at specific sites of interest were removed to create the corresponding radical species. All resulting structures were then optimized using Gaussian 09 ^41^ with the Becke, 3-parameter, Lee–Yang–Parr (B3LYP) functional^42,43^ and the 6-311+G­(d,p) basis set. Bond dissociation energies were subsequently calculated based on the optimized structures, following

$${\Delta E}_{\left(C-H\right)/\left(N-H\right)}=E_{\left(C-H\right)/\left(N-H\right)}-E_{C^{•}/N^{•}}-E_{H^{•}}$$

## Results and Discussion

### Sorbent Characterization—CO_2_ Capacity

Following the synthesis of each sorbent, the CO_2_ uptake of the fresh sorbents was measured. As shown in Figure 2, the incorporation of the additives results in reduction of the CO_2_ uptake for all sorbents. The reduction in the level of CO_2_ uptake is expected, as the incorporation of the additives can impact the rate of CO_2_ diffusion or prevent/block some amine sites from being accessible for CO_2_ adsorption. Accordingly, the sorbent with the bulkiest additive (BDDPA/PEI/Al_2_O_3_) showed the lowest CO_2_ uptake compared with the other candidates.

**Figure 2:**
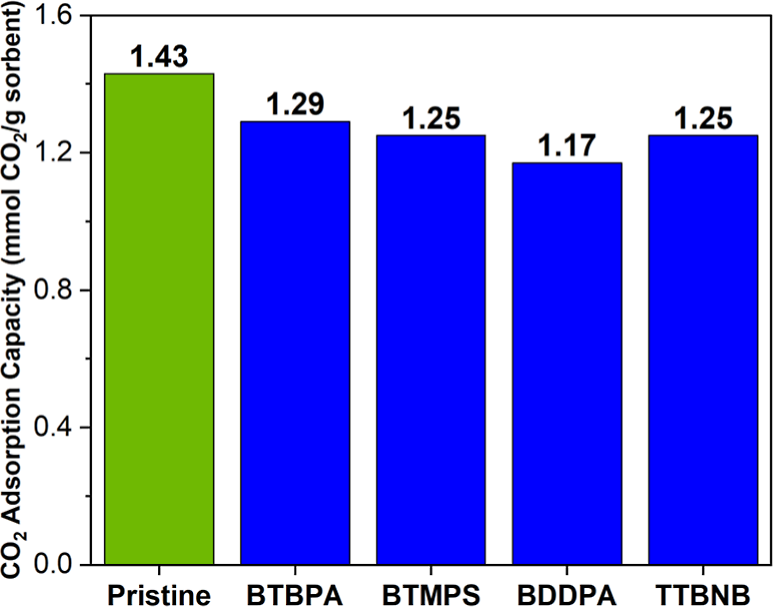
Initial CO_2_ adsorption capacity of sorbents before exposure to oxidative environments (each sorbent incorporates 2 mol % additive, except for the pristine PEI/Al_2_O_3_ sorbent).

### Oxidative Degradation

Oxidative degradation experiments were conducted to evaluate the performance of each sorbent incorporating additives compared with the pristine sorbent performance under the same conditions. Each sorbent was exposed to dry CO_2_-free air (21% O_2_/balance-N_2_) during the oxidative degradation experiment for 4.5 h at 120 °C. After the oxidative treatment, the CO_2_ adsorption capacity of the oxidized sorbents was measured to determine the impact of oxidation.

As the results in Figure 3 show, the mass loss during the oxidative treatment was not significant (<5%) for all sorbents. However, the differences in sorbent deactivation among most of the sorbents were considerable. The sorbents with the lowest mass loss (BTMPS, BTBPA, and BDDPA/PEI/Al_2_O_3_) show the lowest deactivation. This relationship suggests that the sorbents are losing key active sites (amines), even though the sorbent mass losses are not significant. The lost mass is most likely due to the bonds of the PEI and/or the additives cleaving due to thermal stress and oxidative degradation reactions, forming gas-phase products.^15,44^

**Figure 3:**
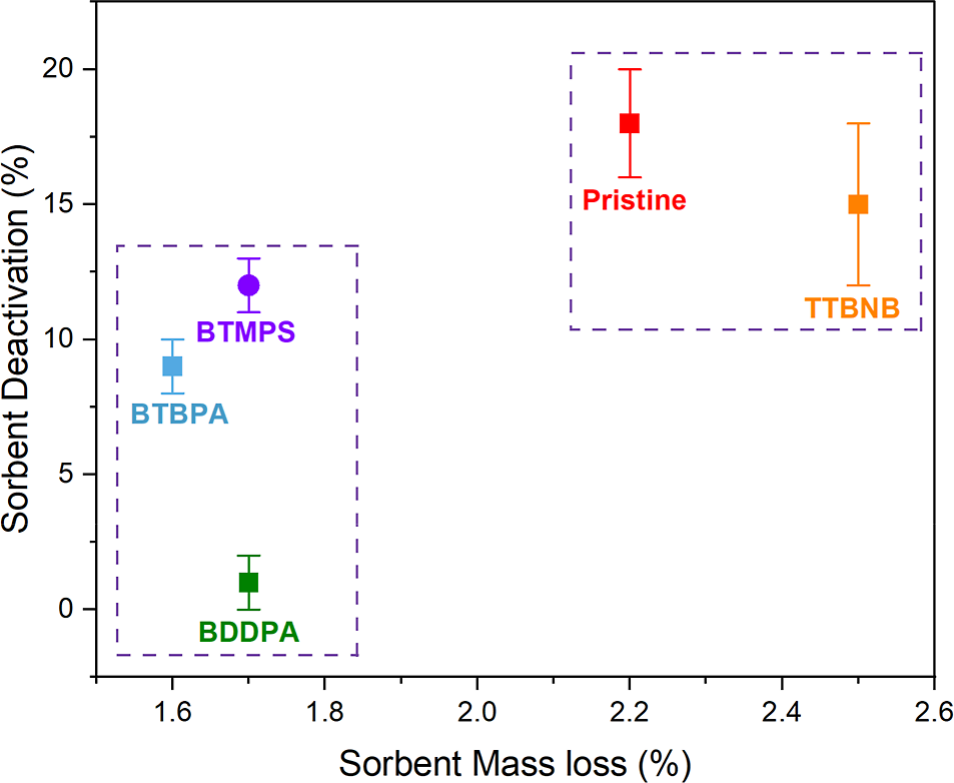
Sorbent mass loss as a function of sorbent deactivation of PEI/Al_2_O_3_ and BDDPA, BTBPA, BTMPS, and TTBNB incorporating PEI/Al_2_O_3_ sorbents after exposure to exposed to CO_2_-free air (21% O_2_/balance-N_2_) for 4.5 h at 120 °C. The error bars indicate the standard deviation of sorbent deactivation based on three replicate runs of experiments.

Among the sorbents explored, the BDDPA/PEI/Al_2_O_3_ sorbent achieves the lowest sorbent deactivation (1%) and the lowest mass loss (1.6%) after continuous exposure to an oxidative environment for 4.5 h. This result suggests that 4,4′-bis­(α,α-dimethylbenzyl)­diphenylamine (BDDPA) is the most effective in inhibiting oxidative degradation reactions among the candidates explored, meriting further study.

To observe the molecular-level changes in the sorbent during the oxidative degradation process, in situ DRIFTS-IR spectroscopy experiments were conducted using the pristine and BDDPA-incorporated sorbents. As Figure 4a shows, the in situ DRIFTS spectra during the oxidation of the BDDPA/PEI/Al_2_O_3_ sorbent show a peak around 1660 cm^–1^, indicating the formation of carbonyl/imine species and a new primary amine species around 1600 cm^–1^.^45,46^ Correspondingly, during the oxidation of the pristine (PEI/Al_2_O_3_) sorbent, similar degradation products (carbonyl/imine species around 1660 cm^–1^ and new primary amine species around 1600 cm^–1^)^18,19,45,46^ formed, as shown in Figure 4b. Despite the similarity in degradation products, the intensity of these peaks (∼1660 cm^–1^ and ∼1600 cm^–1^) was much higher during the oxidation of the pristine sorbent than that of the BDDPA/PEI/Al_2_O_3_ sorbent. The integrated peak areas of the 1660 cm^–1^ and 1600 cm^–1^ bands shown in Figure S2a,b, respectively, also illustrate the difference in the amount of products formed between the two sorbents. The evolution of the 1660 cm^–1^ and 1600 cm^–1^ peaks in the in situ DRIFTS-IR analysis suggests that BDDPA reduces the level of formation of oxidative degradation products. The comparisons in peak intensity and integrated area were made by normalizing the spectra using the γ-Al_2_O_3_ Al–O peak (∼935 cm^–1^)^47^ as a reference, as it remained unchanged throughout the degradation process (Figure S3).

**Figure 4:**
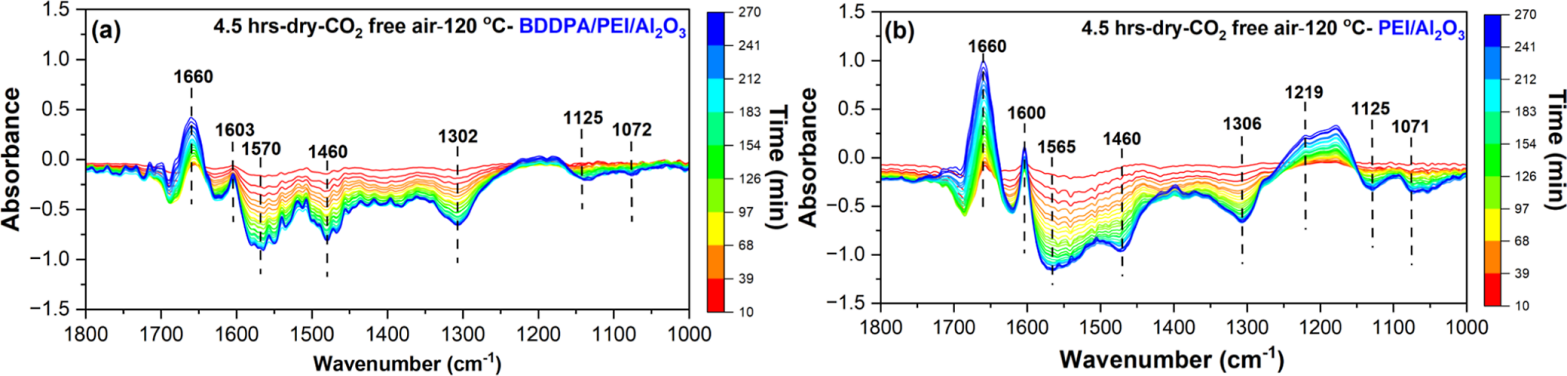
DRIFTS-IR spectra of (a) BDDPA/PEI/Al_2_O_3_ and (b) PEI/Al_2_O_3_ sorbents under CO_2_-free air (21% O_2_/balance-N_2_) for 4.5 h at 120 °C during 4.5 h of oxidation.

### Oxidation Inhibition Mechanism

In aminopolymer sorbent degradation, carbon-centered radicals such as alkyl radicals can form due to hydrogen abstraction by ppm-level metal impurities in the aminopolymer (PEI) or support material (Al_2_O_3_) or due to thermal stress during heating. Under CO_2_-free air, alkyl radicals can react with oxygen molecules, forming alkyl-peroxyl radicals (ROO^•^), as shown in Scheme 1 (steps i and ii).^17,18,32,48^ During the oxidative degradation of the BDDPA/PEI/Al_2_O_3_ sorbent, the secondary amine, which is akin to the reactive nitrogen in BDDPA, scavenges the alkyl-peroxyl radical (ROO^•^) that forms via the reaction of alkyl radicals (R^•^) and O_2_ molecules present in the CO_2_-free air.^49–52^

**Scheme 1:**
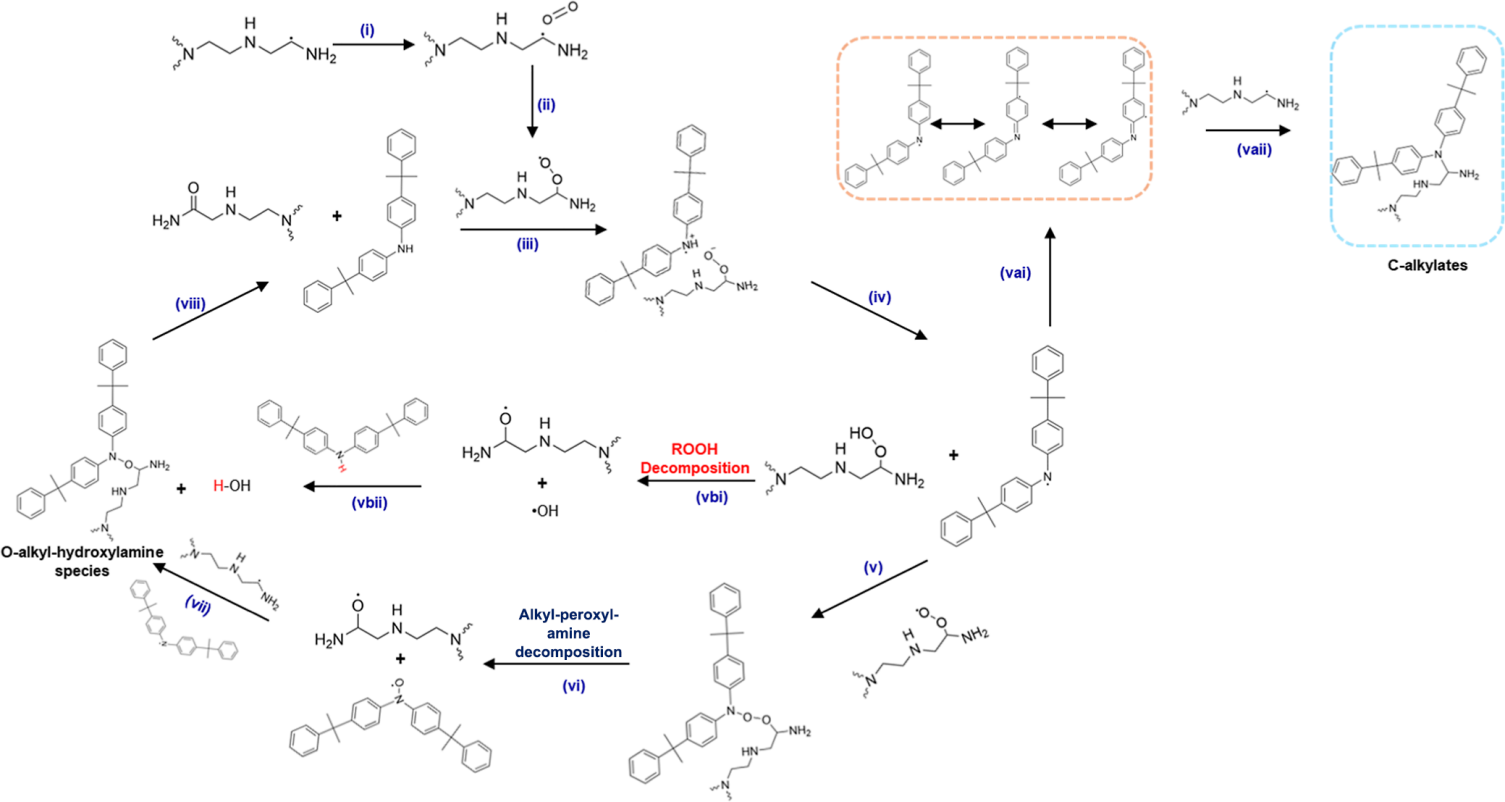
Proposed Mechanism for the Inhibition of PEI/Al_2_O_3_ Sorbent Using 4,4′-Bis­(α,α-dimethylbenzyl)­diphenylamine (BDDPA)^a^

a Adapted with permission from ref 74. Copyright 2012 American Chemical Society.

BDDPA is a kind of diarylamine known as a radical-trapping antioxidant (RTA). RTAs are chain-breaking antioxidants that trap chain-carrying radicals, such as alkyl-peroxyl radicals, to prevent subsequent oxidative degradation reactions. Generally, diarylamines actively inhibit the oxidation of organic materials, specifically hydrocarbons.^49,51–56^ The alkyl-peroxyl radical scavenging process begins with an electron transfer from the secondary amine of BDDPA to the alkyl-peroxyl radical, forming a charge-transfer complex (Scheme 1, step iii), followed by hydrogen abstraction, subsequently yielding alkyl-hydroperoxide and diaryl-aminyl radicals, as shown in step iv in Scheme 1.^49,56–58^ Density functional theory calculations show that the N–H bond dissociation energy at the secondary amine site of BDDPA is lower than that at the amines of triethylenetetramine (TETA), a model compound for PEI (Figure S4a,b). Several studies in the literature examining the antioxidant activities of diarylamines, such as BDDPA, have reported that due to resonance stabilization of the resulting diaryl-aminyl radical, the reactive nitrogen species (secondary amines of BDDPA or BTBPA) of diarylamines are more reactive toward alkyl, alkyl-peroxyl, or hydroxyl radicals.^58–62^ As such, even though the amine species of PEI can potentially scavenge free radicals, the secondary amines of BDDPA are more likely to undergo a hydrogen atom transfer (HAT) reaction and donate a hydrogen atom to the alkyl-peroxyl radical, forming the resonance-stabilized diaryl-aminyl radical.^58,63^ Recalling Figure 3, the resonance stabilization of the diaryl-aminyl radicals is one of the primary reasons for the higher oxidative stability of the sorbents incorporating BDDPA and BTBPA compared to the pristine sorbent as well as sorbents incorporating other radical scavengers (BTMPS and TTBNB).

The aromatic structures in BDDPA can also lead to the formation of carbon-centered mesomeric free radicals (Scheme 1, step vai) by delocalization of the diaryl-aminyl radical. These carbon-centered mesomeric free radicals can react with alkyl radicals on a PEI chain, forming *N*-alkylates (or C-alkylates—for the carbon-centered mesomeric free radicals) (Scheme 1, step vaii), or undergo N–N coupling (C–N or C–C coupling—for the carbon-centered mesomeric free radicals), forming products of higher molecular weight than BDDPA.^53^

The diaryl-aminyl radical formed in step iv reacts with an alkyl-peroxyl radical, forming an alkyl-peroxy-amine species in step v, which decomposes into an alkoxyl radical and diaryl-aminoxyl radical (a more stable radical)­(step vi in Scheme 1). The diaryl-aminoxyl radical proceeds to scavenge an alkyl radical in step vii, forming an *O*-alkyl-hydroxylamine (alkoxyl-amine) species.^52,59,60^ Similarly, the alkyl-hydroperoxide formed in step iv can also lead to the formation of *O*-alkyl-hydroxylamine (alkoxyl-amine) species via the reaction of the decomposition free radicals, a hydroxyl radical (^•^OH) and an alkoxyl radical (R–O^•^)^64^ (step vbi), with the hydroxyl radical abstracting a hydrogen atom from a BDDPA molecule to form an H_2_O molecule and the resulting diaryl-aminyl radical reacting with the alkoxyl radical (step vbii).^49,52,59,61,65,66^ Subsequently, the N–O bond of the *O*-alkyl-hydroxylamine species dissociates and undergoes in-cage disproportionation to regenerate the BDDPA (diarylamine) and forms an amide species (step viii).^52,59,60,65,67^ This regeneration of the diarylamine is one of the apparent reasons for the high oxidative stability and efficiency of BDDPA. A key point in this process is that the diarylamine regeneration step requires high temperatures to provide sufficient thermal energy and break the N–O bond. Hence, the antioxidant activity of BDDPA (and diarylamines) is higher at elevated temperatures (>100 °C).^52,59,60^

As thermal stability is necessary to undergo multiple regeneration cycles, the overall thermal stability of diarylamine can contribute to prolonged antioxidant activity. To that end, the observable difference in inhibiting sorbent deactivation in Figure 3 between BDDPA- and BTBPA-incorporating sorbents under CO_2_-free air could be due to BDDPA having higher thermal stability than BTBPA, which lacks the α,α-dimethylbenzyl groups. To test this, the thermal stabilities of BDDPA and BTBPA were compared. Figure S5 confirms that BDDPA has higher thermal stability than BTBPA. In addition to providing thermal stability, the α,α-dimethylbenzyl groups can extend the lifetime of BDDPA by shielding the diaryl-aminyl radical from other oxidizing agents or preventing undesired side reactions of the diphenyl groups of BDDPA.^52,68,69^ Another factor contributing to the lower performance of the BTBPA incorporating sorbent might be the presence of impurities in the BTBPA additive that reduce the antioxidative activity, such as trace metals and/or organic contaminants. The reported purities of BTBPA and BDDPA were >90% and 98%, respectively, as reported by the respective vendors.

As shown in the proposed mechanism in Scheme 1, BDDPA inhibits oxidative degradation by trapping free radicals and continuously regenerating itself. However, there is a possibility of side reactions occurring that slow the inhibition process. These side reactions could involve the oxidation of the diaryl-aminyl radical, forming quinone-imine species, or the nitroxide (diaryl-aminoxyl radical) to form quinone-nitrone species, with both reactions cleaving an aromatic group in the process.^52,70^ It is important to note that the side reactions can also occur with BTBPA (cleaving the C–C bond of the *tert*-butyl group and forming the carbonyl of quinone-imine or nitrone species), possibly with more ease compared to BDDPA, as the α,α-dimethylbenzyl groups can provide steric hindrance to reduce the side reactions from occurring.^68,69^

The other candidate is BTMPS, which is a hindered amine stabilizer (HAS). HAS are radical-trapping agents with a base molecule of 2,2,6,6-tetramethylpiperidine. They reduce the rate of radical propagation reactions by trapping oxygen- and carbon-centered free radicals. Similar to diarylamines, the amine species play a critical role in the inhibition process of BTMPS. The secondary amine of BTMPS forms di-*tert*-alkyl-aminyl radical species by donating a hydrogen atom to alkyl or alkyl-peroxyl radicals during oxidative degradation reactions.^49,50,53,71,72^ The di-*tert*-aminyl radical reacts with alkyl-peroxyl radicals (ROO^•^), subsequently decomposing to form di-*tert*-aminoxyl radical (R_2_–N–O^•^), which is a stable nitroxide radical (N–O^•^) and alkoxyl radical (R–O^•^).^52,68,71,73^ Like other HAS molecules, BTMPS follows the Denisov cycle, where the di-*tert*-aminoxyl radical (R_2_–N–O^•^) scavenges alkyl radicals (R^•^) to form alkoxy-amine (N–OR) species. The alkoxy-amine species reacts with the alkyl-peroxyl radical, forming a nonradical carbonyl product, such as amides in the case of PEI, and regenerating the di-*tert*-aminoxyl radical (R_2_–N–O^•^) to continue the Denisov cycle.^52,67,72–74^ The general reaction mechanism of HAS is shown in Scheme S1.^74^

In comparison to amine species in PEI, the secondary amine of BTMPS (or secondary amines of other HAS) is more reactive toward free radicals such as alkyl or peroxyl radicals, hence improving the sorbent’s resistance to oxidative degradation, as shown in Figure 3.^62^ BTMPS provides improved stability due to (i) the steric hindrance provided by the tetramethyl groups to the di-*tert*-aminyl radical formed following the hydrogen atom abstraction and (ii) the regenerable di-*tert*-aminoxyl radical (R_2_–N–O^•^), which continues to participate in the Denisov cycle scavenging free radicals.^52,73^

Both BTMPS and BDDPA contain secondary amines and follow regenerative processes to scavenge free radicals, but their performance shown in Figure 3 is quite different.^52,67,74^ Possible reasons for the observation that BTMPS is less effective than BDDPA in inhibiting oxidative degradation are (i) the lack of free radical delocalization after hydrogen abstraction from BTMPS, (ii) the lower energy barrier to regenerate BDDPA (diaryl-amine species) than the di-*tert*-aminoxyl radical (R_2_–N–O^•^) (nitroxide radical),^65,67^ and (iii) the lower thermal stability of BTMPS compared to that of BDDPA.^75^

Finally, the ineffectiveness of TTBNB in inhibiting the oxidative degradation of PEI/Al_2_O_3_ sorbent shown in Figure 3 could be due to its spin-trapping mechanism of scavenging free radicals, which is not regenerable.^76^ In the spin-trapping mechanism, TTBNB molecules scavenge free radicals via the nitrogen of the nitroso group (N–O), forming a stable nitroxide radical (N–O^•^) adduct.^77^ While this adduct is much more stable than free radicals due to resonance stabilization and steric hindrance, it does not convert back to the initial TTBNB form; as such, it is consumed throughout the degradation process.^76,78^

### Impact of Oxidation Time

To examine the effectiveness of the BDDPA additive over time, the BDDPA/PEI/Al_2_O_3_ sorbent was exposed to CO_2_-free air (21% O_2_/balance N_2_) for 4.5, 9, and 18 h at 120 °C. As illustrated in Figure 5, the performance of the BDDPA/PEI/Al_2_O_3_ sorbent decreased as the exposure time increased. However, the sorbent maintained improved stability compared to the pristine (PEI/Al_2_O_3_) sorbent under all conditions tested. The BDDPA/PEI/Al_2_O_3_ sorbent retained approximately 50% of its capacity after 18 h of exposure, indicating that BDDPA continues to scavenge free radicals actively.

**Figure 5:**
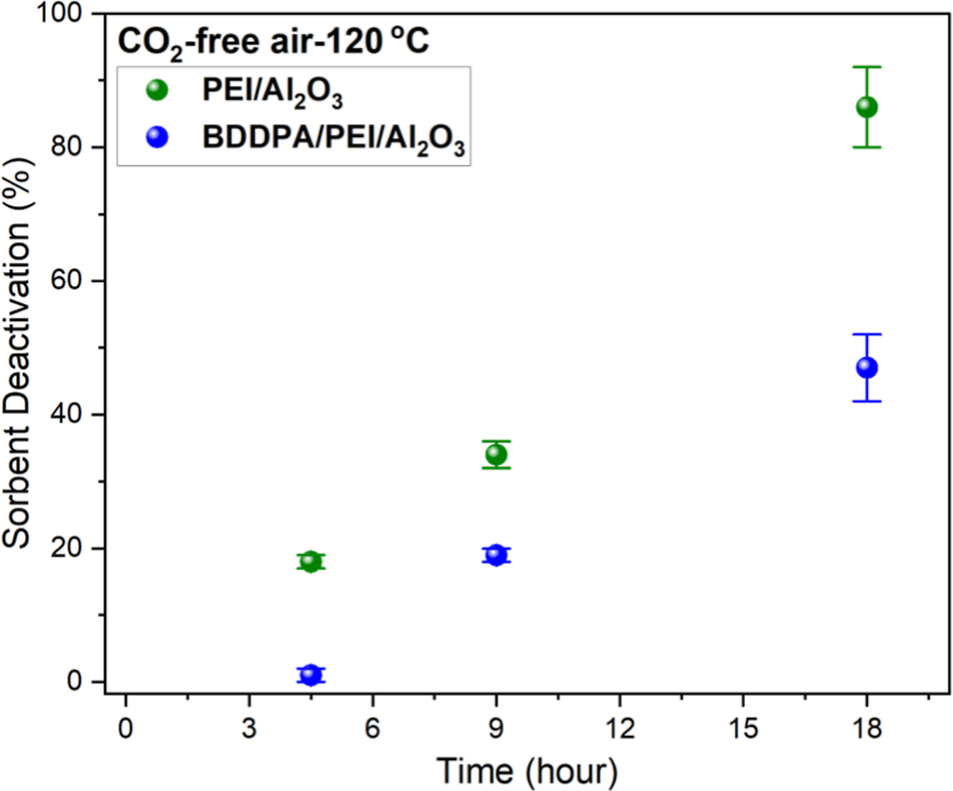
Sorbent deactivation of the pristine (PEI/Al_2_O_3_) and BDDPA/PEI/Al_2_O_3_ sorbents after 4.5, 9, and 18 h of oxidation at 120 °C under 21% O_2_/balance N_2_.

### Impact of BDDPA Additive Loading

To investigate if increased additive loading can enhance the stability of the sorbent further, PEI/Al_2_O_3_ sorbents incorporating 1 and 4 mol % BDDPA were synthesized. The synthesized sorbents were exposed to CO_2_-free air for 4.5 h at 120 °C, and their retained CO_2_ adsorption capacity was compared to that of 2 mol % BDDPA-incorporated PEI/Al_2_O_3_ sorbent. As shown in Figure 6, increasing or decreasing the BDDPA additive loading from 2 mol % to 4 mol % or 1 mol %, respectively, did not enhance the stability of the sorbent. Improved stability was not observed with increased BDDPA content (4 mol % BDDPA-containing sorbent), maybe due to lower BDDPA molecule dispersion throughout the sorbent, leading to reduced protection against oxidation if the BDDPA molecules are clustered together.

**Figure 6:**
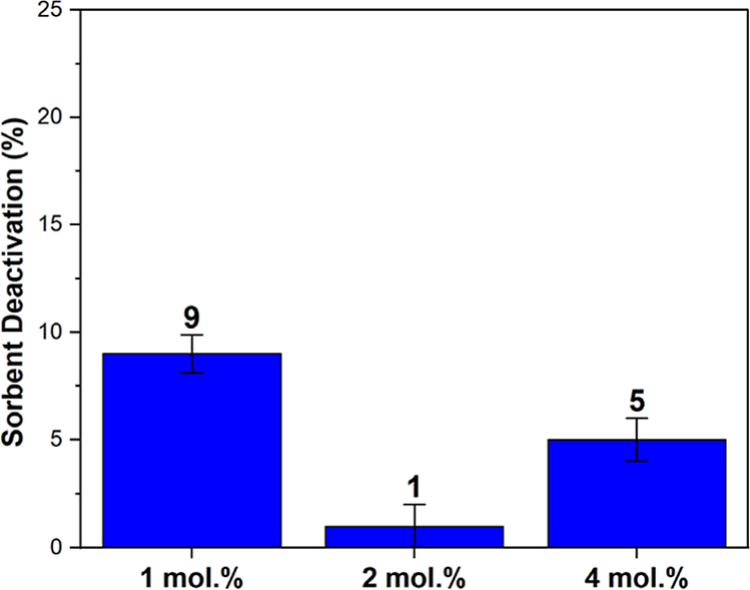
Sorbent deactivation after 4.5 h of oxidation at 120 °C under CO_2_-free air for 1, 2, and 4 mol % BDDPA additive incorporated PEI/Al_2_O_3_ sorbents. The error bars are determined by performing two sorbent deactivation experiments.

Based on the results collected thus far under the CO_2_-free oxidative conditions, the 2 mol % BDDPA/PEI/Al_2_O_3_ sorbent performs better and shows improved stability. As such, the (2 mol % BDDPA/PEI/Al_2_O_3_) sorbent was selected for further investigation employing various temperatures and gas mixtures used in DAC processes. As reported in recent studies on the stability of PEI/Al_2_O_3_, two key species in air, CO_2_ and H_2_O, play a significant role in influencing sorbent lifetime.^17–19^ Accordingly, the stability of the 2 mol % BDDPA/PEI/Al_2_O_3_ sorbent is explored under conditions that include 0.04% CO_2_ and/or H_2_O.

### Impact of CO_2_-Containing Air

Adsorbed CO_2_ species under 0.04% CO_2_-air and temperatures above 55 °C can catalyze sorbent degradation via C–N bond cleavage reactions.^17^ As such, the BDDPA/PEI/Al_2_O_3_ sorbent was exposed to dry 0.04% CO_2_-air (21% O_2_ balance N_2_) for 4.5 h at 120 °C to investigate the stability of the sorbent under CO_2_-containing air. As shown in Figure 7a, the BDDPA/PEI/Al_2_O_3_ sorbent shows enhanced stability, similar to the result obtained under dry CO_2_-free conditions. While the obtained result under CO_2_-containing air signifies the effectiveness of BDDPA in enhancing stability at 120 °C, CO_2_ minimally contributes to the degradation process at this temperature. Indeed, under 0.04% CO_2_-air, oxidative and thermal degradation reactions dominate at temperatures above 100 °C, whereas CO_2_-catalyzed degradation reactions are important below 100 °C. This is due to the reduction in the surface CO_2_ loading and the faster rate of oxidative and thermal reactions at higher temperatures.^17^ To better observe the impact of adsorbed CO_2_, the sorbent was exposed to 0.04% CO_2_-air for 4.5 h at 70 °C, where CO_2_–amine interactions occur more strongly compared to 120 °C.^79^ At 70 °C, both sorbents were more stable than at 120 °C, and the difference in deactivation between the two sorbents was reduced compared to 120 °C. Nevertheless, the BDDPA/PEI/Al_2_O_3_ sorbent performed better than the pristine sorbent, as illustrated in Figure 7b. The results in Figure 7a,b show that BDDPA participates in resisting degradation under both temperatures explored, suggesting that the cyclic radical inhibition mechanism (Scheme 1) still holds even at temperatures below 100 °C.

**Figure 7:**
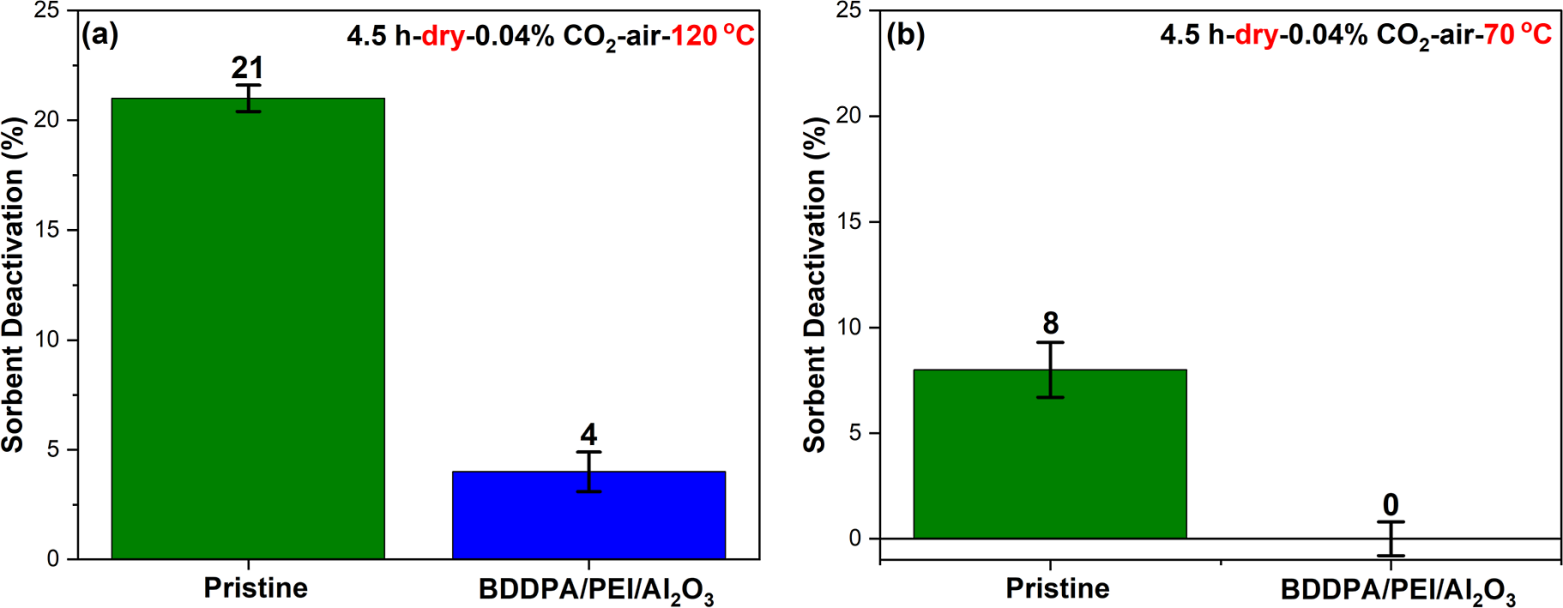
Sorbent deactivation of the pristine and BDDPA/PEI/Al_2_O_3_ sorbent after 4.5 h under dry 0.04% CO_2_-air at (a) 120 °C and (b) 70 °C.

At an extended exposure time of 18 h (70 °C), both sorbents sustained substantial degradation, but the BDDPA/PEI/Al_2_O_3_ sorbent performed better (Figure S6), as it did after 4.5 h of exposure, suggesting BDDPA actively participates in the radical scavenging process even at an extended exposure time under 0.04% CO_2_-air. The higher hydrogen loss (in comparison to carbon and nitrogen) in the C, H, and N analysis results of the BDDPA/PEI/Al_2_O_3_ sorbent under 0.04% CO_2_-air at 70 °C (Figure S7a) supports the hypothesis that BDDPA stabilizes the sorbent by donating hydrogen atoms to the aminyl and alkyl radicals formed during CO_2_-induced C–N bond cleavage reactions as well as oxidative and thermal degradation reactions. Figure S7b shows that the pristine sorbent lost more of its C, H, and N contents in comparison to the BDDPA/PEI/Al_2_O_3_ sorbent, further supporting the higher sorbent deactivation in Figure S6.

The increased deactivation from 0% after 4.5 h to 30% after 18 h is most likely due to enhanced radical species formation (alkyl, peroxyl, aminyl) originating from the C–N bond cleavage reactions catalyzed by CO_2_ leading to accelerated sorbent degradation,^17,19^ overwhelming BDDPA molecules, which predominantly inhibits alkyl and peroxyl radicals^17,19,49,51,57,80^ Another possible phenomenon that might have contributed to the enhanced deactivation is the interaction of the secondary amine of BDDPA with CO_2_, leading to the inability of BDDPA to scavenge radicals. However, CO_2_ adsorption experiments using only BDDPA, in the absence of PEI, show that there is no significant CO_2_ adsorption occurring (Figure S8).

### Impact of Humidity (in CO_2_-Free and CO_2_-Containing Air)

Like the contrasting impact of CO_2_ on aminopolymer sorbent stability, H_2_O has been reported to both enhance sorbent stability and accelerate sorbent degradation.^18,19,81^ It is hypothesized that H_2_O vapor can enhance sorbent degradation (i) physically by increasing aminopolymer mobility, which can allow increased O_2_ diffusion and subsequent O_2_ attack^18^ and (ii) chemically by contributing to the formation of ^•^OH radicals, which catalyze sorbent degradation,^18^ and/or by catalyzing the C–N bond cleavage reaction.^17^

In this study, the stability of a BDDPA/PEI/Al_2_O_3_ sorbent was investigated under humid (∼43% RH at 26 °C) CO_2_-free air and 0.04% CO_2_-air environments at 120 and 70 °C for 4.5 h. First, humid oxidation experiments were conducted at 120 °C for 4.5 h to complement the dry CO_2_-free air experiments under the same conditions. As depicted in Figure 8, while both sorbents showed higher deactivation compared to the dry conditions (Figure 5), the BDDPA/PEI/Al_2_O_3_ sorbent showed improved stability compared to the pristine sorbent. The enhanced stability under humid conditions indicates that BDDPA continues to react with/trap radicals, reducing sorbent degradation and the formation of degradation products.

**Figure 8:**
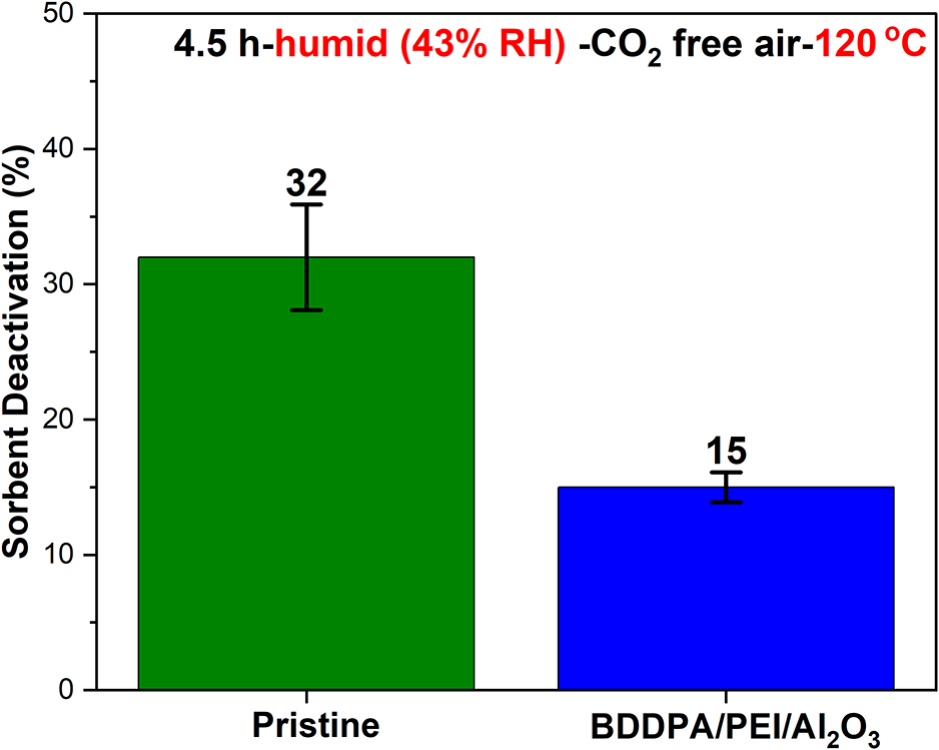
BDDPA PEI/Al_2_O_3_ and the pristine sorbent deactivation after 4.5 h under humid (∼43% RH at 26 °C) CO_2_-free air at 120 °C.

Similar to the dry condition, the in situ DRIFTS spectra of both sorbents under humid CO_2_-free air at 120 °C show the formation of degradation products such as carbonyl/imine species around 1674 cm^–1^ and new primary amine species around 1600 cm^–1^.^18,19,45,46^ However, the BDDPA/PEI/Al_2_O_3_ sorbent spectra show less formation of degradation products due to the reduced sorbent deactivation compared to the pristine sorbent (Figure 9a,b). The integrated peak area of the carbonyl/imine and primary amine species for the BDDPA/PEI/Al_2_O_3_ and pristine sorbents further confirms the decreased formation of degradation products (Figure S9a,b). The reduction in effectiveness under humid conditions can be attributed to (i) enhanced PEI mobility, leading to more frequent interactions of radicals (alkyl, aminyl, or alkoxyl) on the PEI backbone and chain with O_2_ molecules, (ii) increased production of ^•^OH radicals from H_2_O molecules, and (iii) water lowering the free energy barrier for C–N bond cleavage reactions.^17,18^

**Figure 9:**
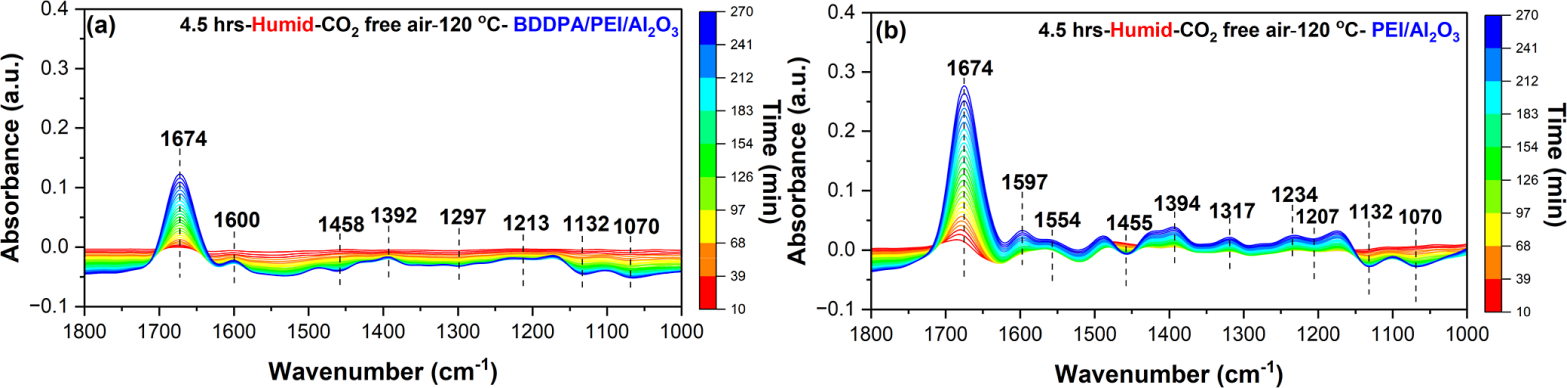
DRIFTS-IR spectra of (a) BDDPA/PEI/Al_2_O_3_ and (b) PEI/Al_2_O_3_ sorbents under humid (∼43% RH at 26 °C) CO_2_-free air (21% O_2_/balance-N_2_) for 4.5 h at 120 °C during 4.5 h of oxidation.

Interestingly, under humid 0.04% CO_2_-air environments, the BDDPA/PEI/Al_2_O_3_ sorbent showed significantly improved stability both at 120 and 70 °C with no substantial loss in CO_2_ adsorption capacity after 4.5 h, as illustrated in Figure 10a,b. On the contrary, the pristine sorbent sustained higher deactivation under humid conditions compared to the dry conditions (Figure 7a,b). Under the humid conditions explored, the BDDPA/PEI/Al_2_O_3_ sorbent showed higher resistance to degradation in the presence of CO_2_ (0.04%) (compared to CO_2_-free air), indicating H_2_O vapor’s reduced activity/role in accelerating sorbent degradation in the presence of CO_2_. The enhanced stability due to the incorporation of BDDPA under dry and humid 0.04% CO_2_-air is promising in prolonging the sorbent lifetime in realistic DAC cyclic processes, as the 0.04% CO_2_-air gas mixture is close to the realistic atmospheric air composition.

**Figure 10:**
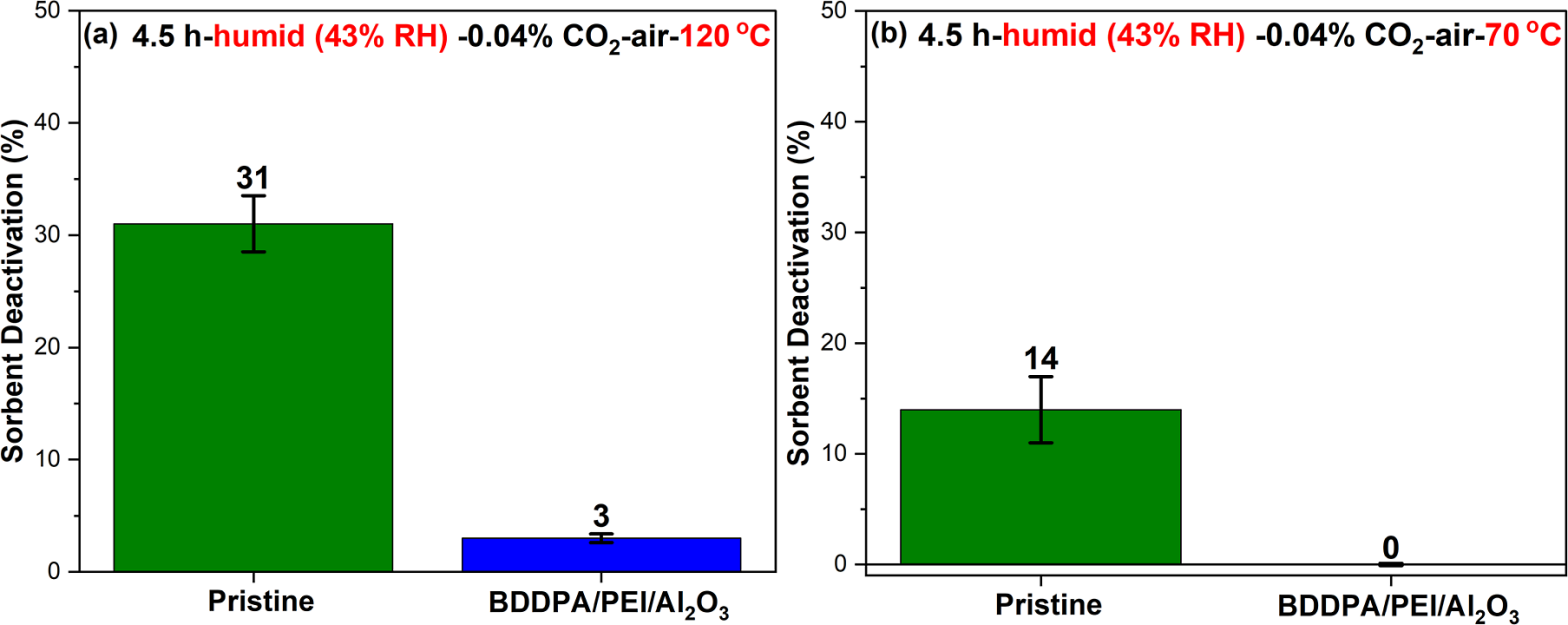
BDDPA PEI/Al_2_O_3_ and the pristine sorbent deactivation after 4.5 h under humid (∼43% RH at 26 °C) 0.04% CO_2_-air at (a) 120 °C (b) and 70 °C.

After 18 h of extended exposure at 70 °C under humid 0.04% CO_2_-air (Figure S10), the stability of both sorbents declined notably compared to the dry conditions (Figure S6). This increased sorbent deactivation could be due to the enhanced formation of radical species as a consequence of the extended exposure and the physical and chemical influences of water molecules discussed under humid CO_2_-free conditions, which can continuously promote sorbent degradation. Despite the increase in deactivation compared to the dry condition, the BDDPA/PEI/Al_2_O_3_ sorbent showed significant improvement in stability in comparison to that of the pristine sorbent. Similar to the dry condition, a higher loss in hydrogen content (in comparison to carbon and nitrogen) was observed for the BDDPA/PEI/Al_2_O_3_ sorbent under humid 0.04% CO_2_-air at 70 °C (Figure S11), further indicating the hydrogen abstraction as one of the key mitigating factors during the degradation process.

## Conclusions

The efficacy of commonly used hydrocarbon polymer stabilizers in mitigating the degradation of aminopolymer sorbents under temperatures and gas mixtures used in DAC processes was explored using the PEI/Al_2_O_3_ sorbent. Except for 2,4,6-tri*tert*-butylnitroso-benzene (TTBNB), all radical scavengers and additives showed improved stability under dry oxidative degradation conditions (CO_2_-free air at 120 °C) compared with the pristine sorbent. The 4,4′-bis­(α,α-dimethylbenzyl)­diphenylamine (BDDPA) incorporated PEI/Al_2_O_3_ sorbent showed the most improvement in stability. The sorbent’s performance at 120 °C under dry CO_2_-free air was replicated at 120 and 70 °C under dry and humid 0.04% CO_2_-air conditions, indicating the antioxidant activity of BDDPA applies to CO_2_-induced degradation reactions and H_2_O-enhanced degradation phenomenon. Under humid CO_2_-free air conditions, the activity of BDDPA in inhibiting degradation reduces (in comparison to the dry CO_2_-free air and humid 0.04% CO_2_-air); however, it shows significant improvement in comparison to the pristine sorbent. The effectiveness of BDDPA in enhancing sorbent stability is proposed to be its ability to regenerate itself in a cyclic radical scavenging process, where it neutralizes alkyl, aminyl, alkoxyl, and hydroxyl radicals during the degradation process. Humidity is a key parameter in DAC processes, impacting sorbent stability and CO_2_ adsorption, and this study demonstrates the effectiveness of BDDPA under humid conditions.

In comparison to other additives, such as sulfur-containing antioxidants reported to enhance oxidative stability of solid amine sorbents, the antioxidative mechanism of BDDPA differs. While BDDPA scavenges free radicals such as alkyl, hydroxyl, and alkyl-peroxyl radicals, forming amide species and regenerating itself in a cyclic process, sulfur-containing additives decompose hydroperoxides that form during the oxidation to alcohols and form sulfides or thio-sulfinates. As such, exploring the synergistic effects of diarylamines, such as BDDPA and sulfur-containing additives, may result in an improved oxidative stability under temperatures and gas mixtures used in DAC processes.

Overall, this work identifies a sorbent-additive composite effective in mitigating oxidative degradation of the PEI/Al_2_O_3_ sorbent at elevated and intermediate temperatures relevant to sorbent regeneration and under dry and humid conditions. Furthermore, it highlights the importance of considering the impact of key environmental components such as CO_2_ and H_2_O on aminopolymer stability and performance when designing degradation-mitigating solutions for DAC applications.

## Supplementary Material



## Notes

Pore volume and surface area values of the support and adsorbents, organic content in each sorbent, ^13^C NMR spectra of each sorbent, integrated peak areas during in situ DRIFTS-IR dry oxidation of BDDPA/PEI/Al_2_O_3_ and pristine PEI/Al_2_O_3_ sorbents, DRIFTS-IR spectra of fresh PEI/Al_2_O_3_ sorbent and after 4.5 h dry oxidation, bond dissociation energy of N–H and CH of TETA molecule, thermal decomposition of BTBPA and BDDPA as a function of temperature, scheme of a simplified Denisov cycle, sorbent deactivation data under dry 0.04% CO_2_-air and 18 h, C, H, and N content change for pristine and BDDPA/PEI/Al_2_O_3_ sorbents after 18 h under dry 0.04% CO_2_-air at 70 °C, CO_2_ adsorption capacity of BDDPA and BDDPA/PEI/Al_2_O_3_, integrated peak areas during in situ DRIFTS-IR humid oxidation of BDDPA/PEI/Al_2_O_3_ and pristine PEI/Al_2_O_3_ sorbents, sorbent deactivation data under humid 0.04% CO_2_-air and 18 h, and C, H, and N content change for pristine and BDDPA/PEI/Al_2_O_3_ sorbents after 18 h under humid 0.04% CO_2_-air at 70 °C (PDF)
