# Supplementary material for: Evaluating Autoxidation Radical Scavengers and Additives to Enhance Aminopolymer Sorbent Stability
Source: Energy Fuels. 2025 Dec 2;39(49):23141–52. doi: 10.1021/acs.energyfuels.5c04042 (PMC12703673; doi:10.1021/acs.energyfuels.5c04042)
Supplement: Supplementary file 1 [file ef5c04042_si_001.pdf]

Supporting Information

Evaluating Autoxidation Radical Scavengers and Additives to Enhance Aminopolymer Sorbent Stability

Yoseph A. Guta,[1] Paco Tang,[1] Sichi Li,[2] Jiaqi Zhang,[1] Miles A. Sakwa-Novak,[3] Simon H. Pang,[2] Carsten Sievers,[1]* Christopher W. Jones[1]*

1. *School of Chemical and Biomolecular Engineering, Georgia Institute of Technology, Atlanta, GA 30332, United States*
2. *Materials Science Division, Lawrence Livermore National Laboratory, Livermore, CA 94550, United States*
3. *Virdis Systems, Inc., 2342 Broadway, San Francisco, CA 94115, United States*

*Corresponding authors: carsten.sievers@chbe.gatech.edu; cjones@chbe.gatech.edu;

Table S1. Pore volume and surface area of the support, pristine sorbent and additive-sorbent composite

Sample	BHJ Pore Volume (cm^3/g)	BET Surface Area (m^2/g)
γ-Al_2O_3	0.82	133
35 wt.% PEI/Al_2O_3	0.22	26
BTBPA/PEI/Al_2O_3	0.07	9
BTMPS/PEI/Al_2O_3	0.08	7
BDDPA/PEI/Al_2O_3	0.06	6
TTBNB/PEI/Al_2O_3	0.14	11

Table S2. Weight fraction organic in each sample

Sample	Expected Organic Content (wt. %)	Actual Organic Content (wt. %)	Percent Deviation
BTBPA/PEI/Al_2O_3	39	37.5	-4.0
BTMPS/PEI/Al_2O_3	41	39.2	-4.4
BDDPA/PEI/Al_2O_3	40	40.5	1.3
TTBNB/PEI/Al_2O_3	39	39.4	1.0

The results in **Table S1** show that the synthesized sorbents incorporating the additives (in terms of organic versus inorganic material) were close to their intended organic and inorganic weight percentages, with the greatest absolute error being 4.4% for BTMPS/PEI/Al_2O_3. Since the fractional deviations are small from sample to sample, we assert that we can make accurate comparisons of the effectiveness between the different additives and the pristine (additive-free) sorbent.

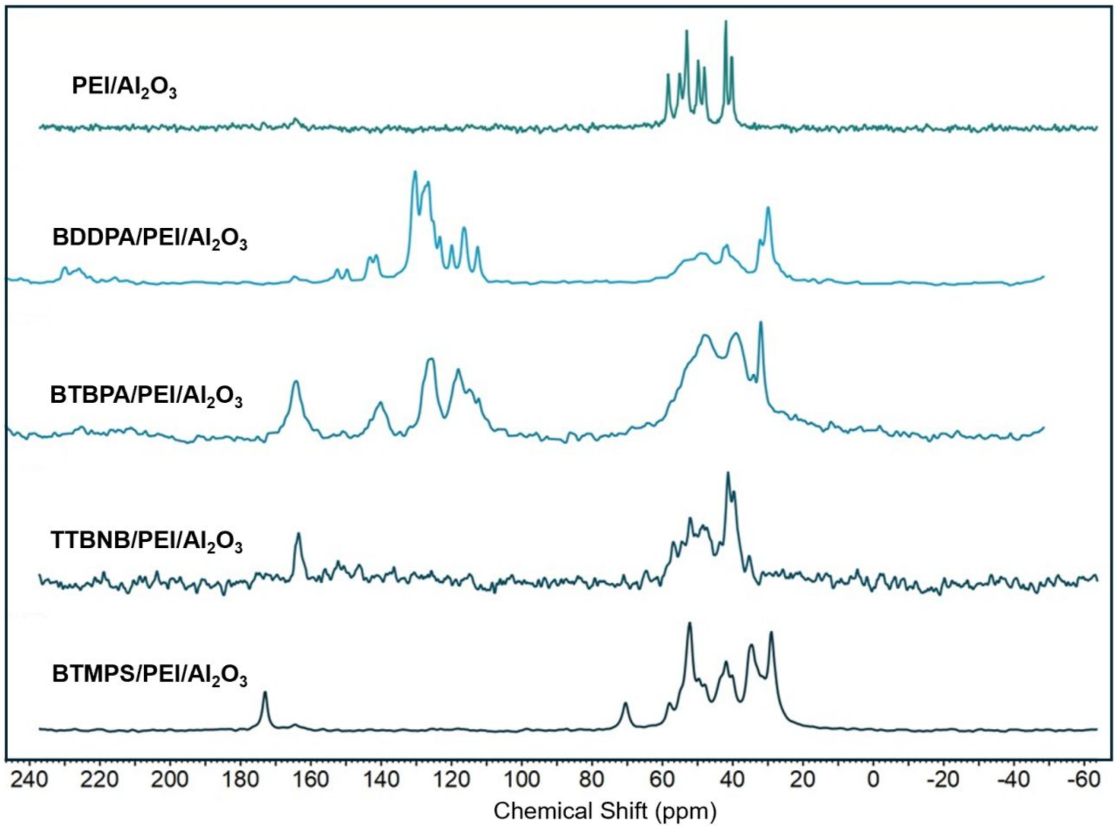

Figure S2. ^{13}C NMR spectra for pristine PEI/Al_2O_3 sorbent and PEI/Al_2O_3 sorbent incorporating BDDPA, BTBPA, TTBNB, and BTMPS additives.

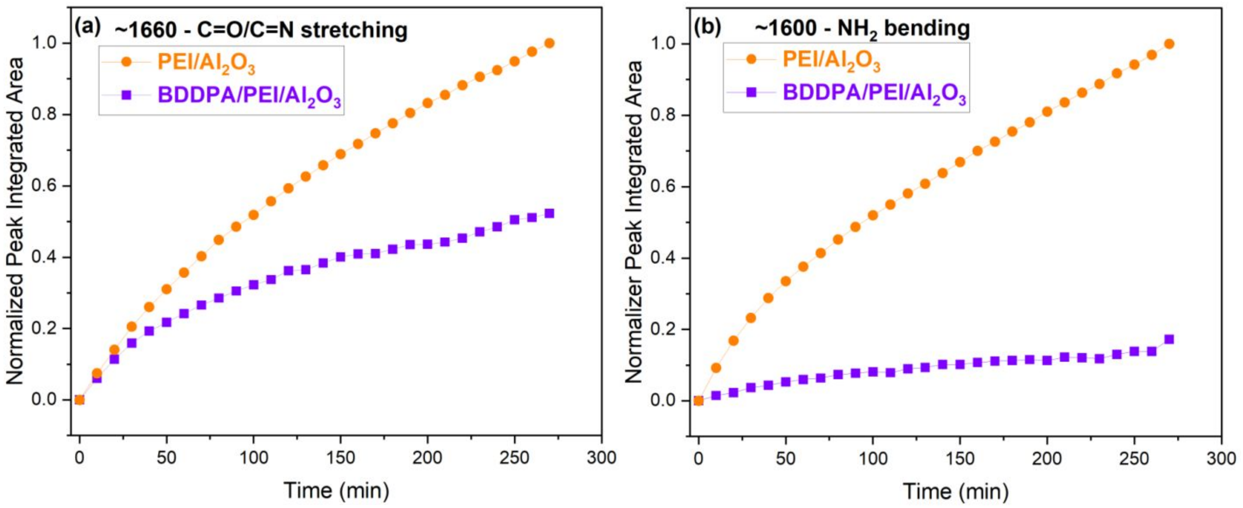

Figure S2. Integrated peak areas of (a) ~1660 cm^{-1} and (b) ~1600 cm^{-1} bands as a function of time during *in situ* DRIFTS-IR oxidation of $BDDPA/PEI/Al_2O_3$ and pristine PEI/Al_2O_3 sorbents under CO_2-free air (21% O_2 /balance-N_2) for 4.5 hours at 120 °C

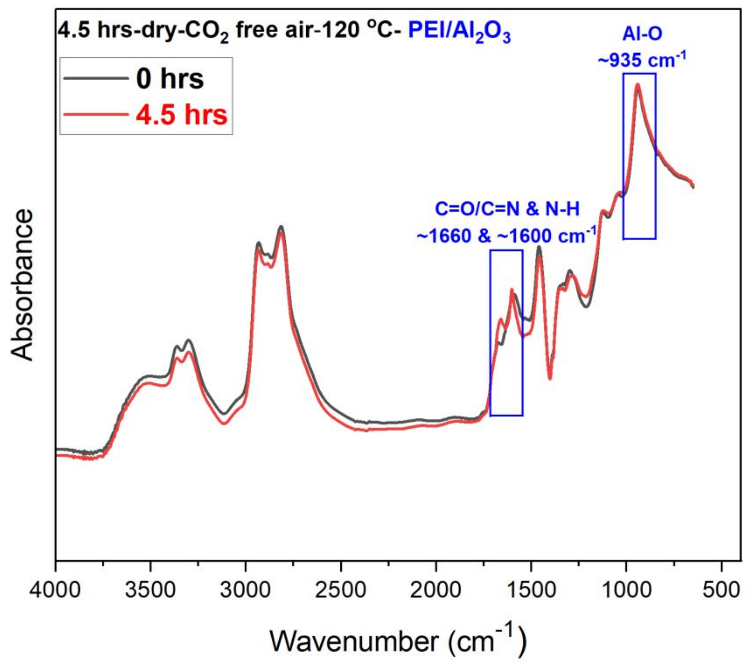

Figure S3. DRIFTS-IR spectra of PEI/Al_2O_3 sorbent under CO_2-free air (21% O_2 /balance-N_2) at 120 °C at time 0 and 4.5 hours.

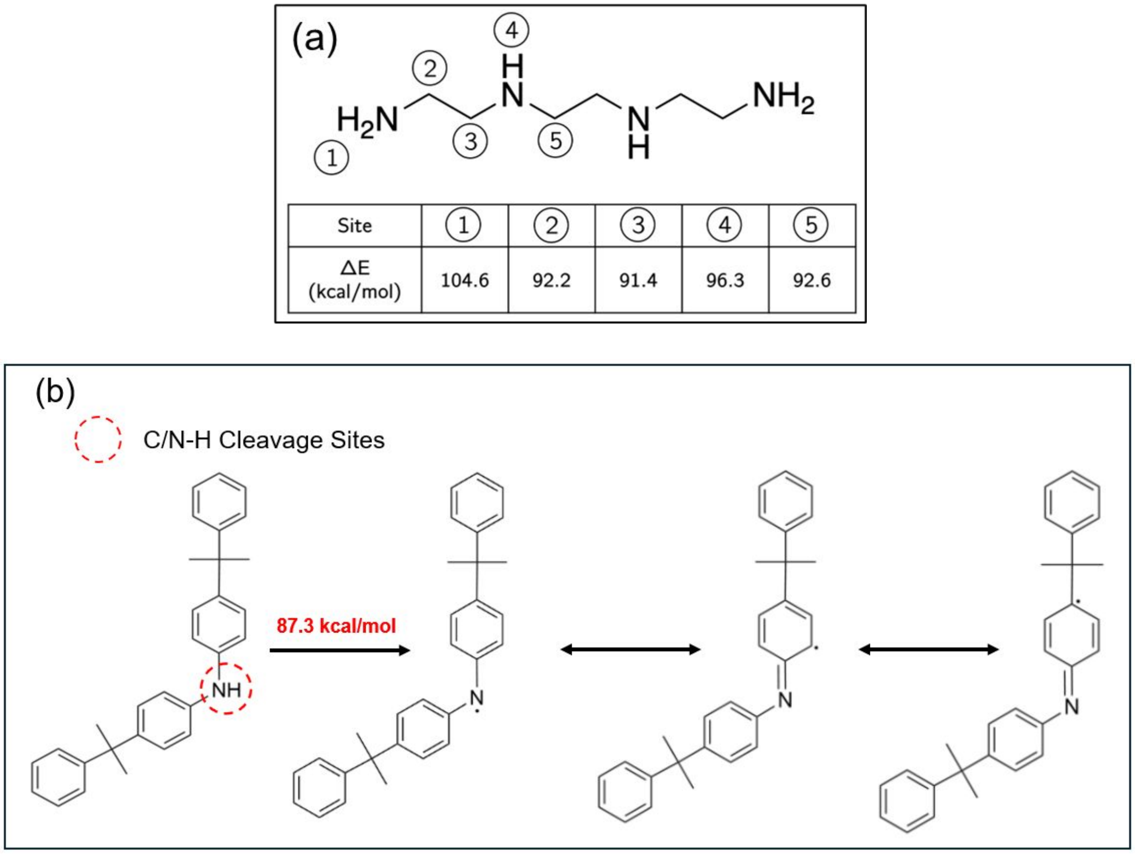

Site	①	②	③	④	⑤
ΔE (kcal/mol)	104.6	92.2	91.4	96.3	92.6

Figure S4. Bond dissociation energy of (a) N-H and C-H of TETA and (b) N-H of BDDPA

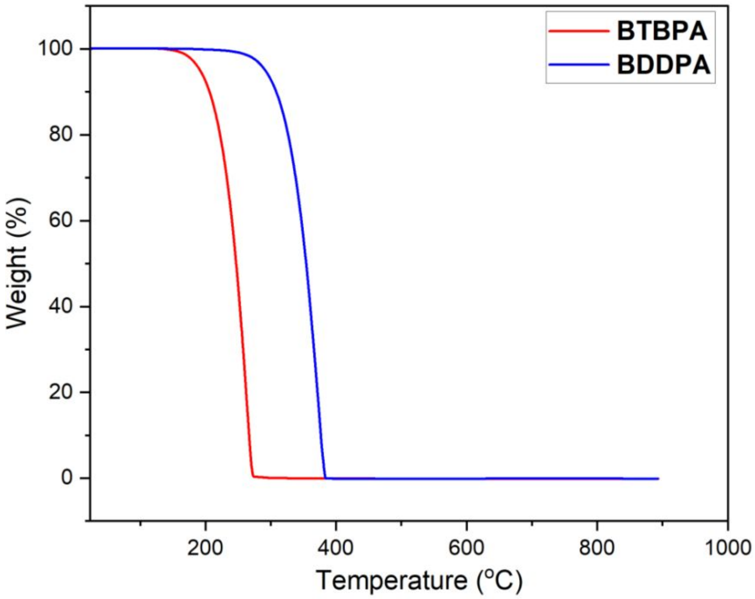

Figure S5. Weight change of BTBPA and BDDPA additives as a function of temperature (under pure N_2)

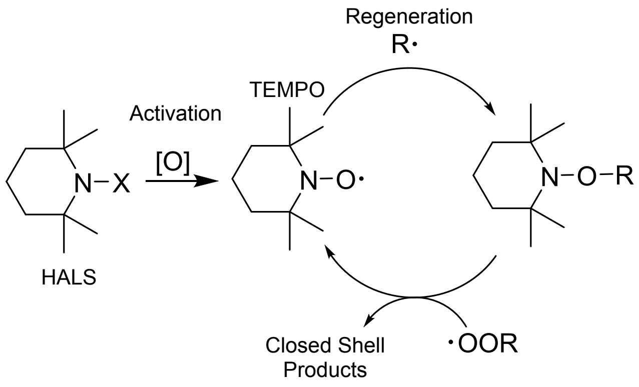

Scheme S1. Simplified mechanism of the Denisov cycle[1] Adapted with permission from [1]. Copyright 2012 American Chemical Society.

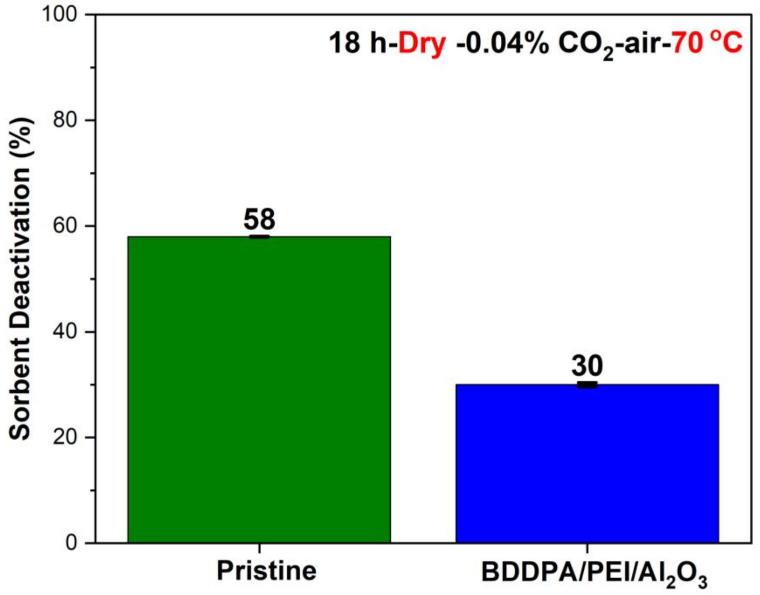

Figure S6. Sorbent deactivation of the pristine and BDDPA PEI/Al_2O_3 sorbent after 18 hours under dry 0.04% CO_2-air at 70 °C

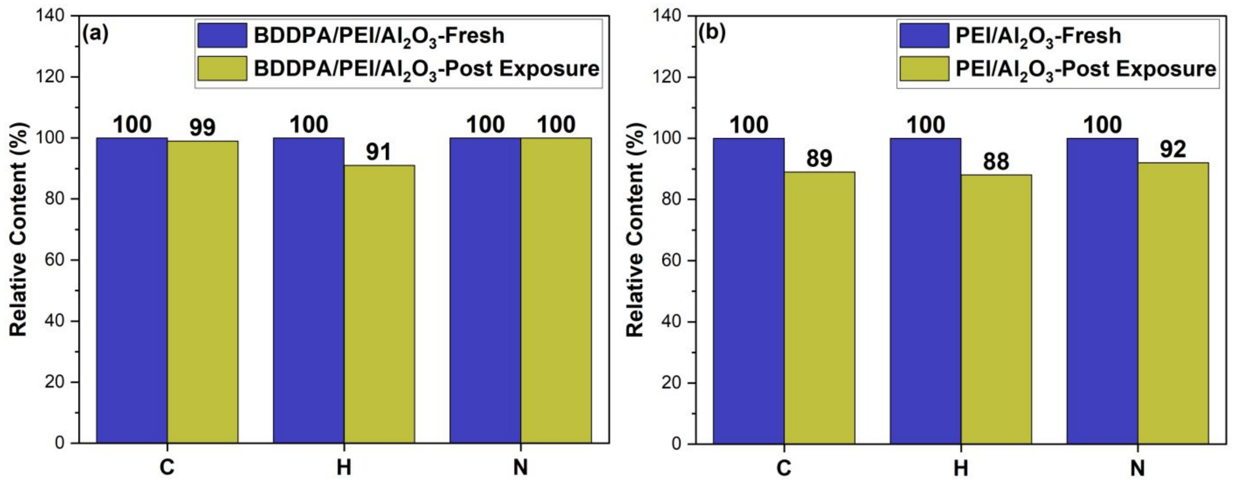

Figure S7. Changes in the carbon, hydrogen, and nitrogen content of the (a) BDDPA/PEI/Al_2O_3; and (b) the pristine (PEI/Al_2O_3) sorbent after 18 hours under 0.04% CO_2-air at 70 °C

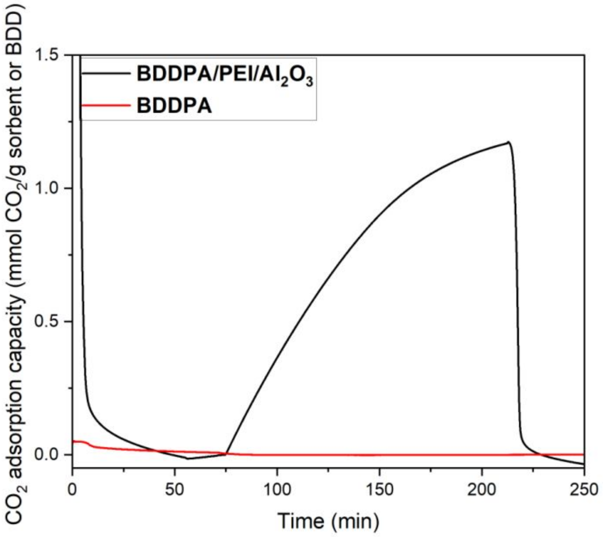

Figure S8. CO_2 adsorption capacity of BDDPA/PEI/Al_2O_2 sorbent and BDDPA under 0.04% CO_2/N_2 at 30 °C

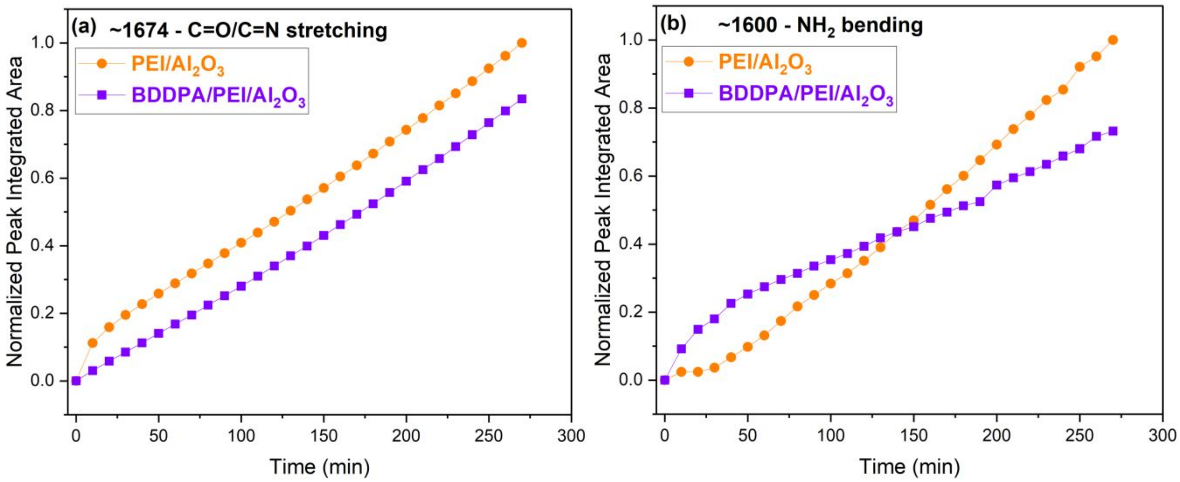

Figure S9. Integrated peak areas of (a) ~1660 cm^{-1} and (b) ~1600 cm^{-1} bands as a function of time during *in situ* DRIFTS-IR oxidation of BDDPA/PEI/Al_2O_3 and pristine PEI/Al_2O_3 sorbents under humid (~43% RH at 26 °C) CO_2-free air (21% O_2 /balance-N_2) for 4.5 hours at 120 °C

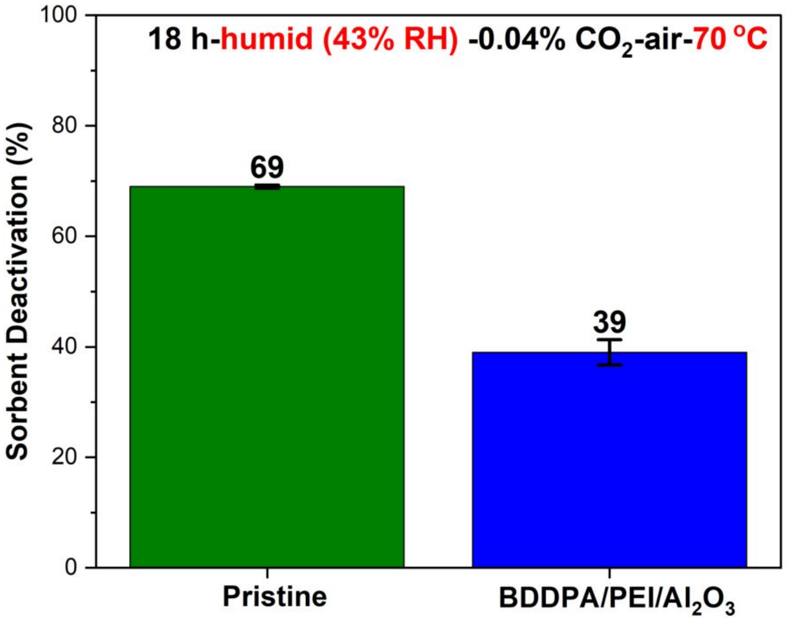

Figure S10. Sorbent deactivation of the pristine and BDDPA PEI/Al_2O_3 sorbent after 18 hours under humid (~43%RH at 26 °C) 0.04% CO_2-air at 70 °C

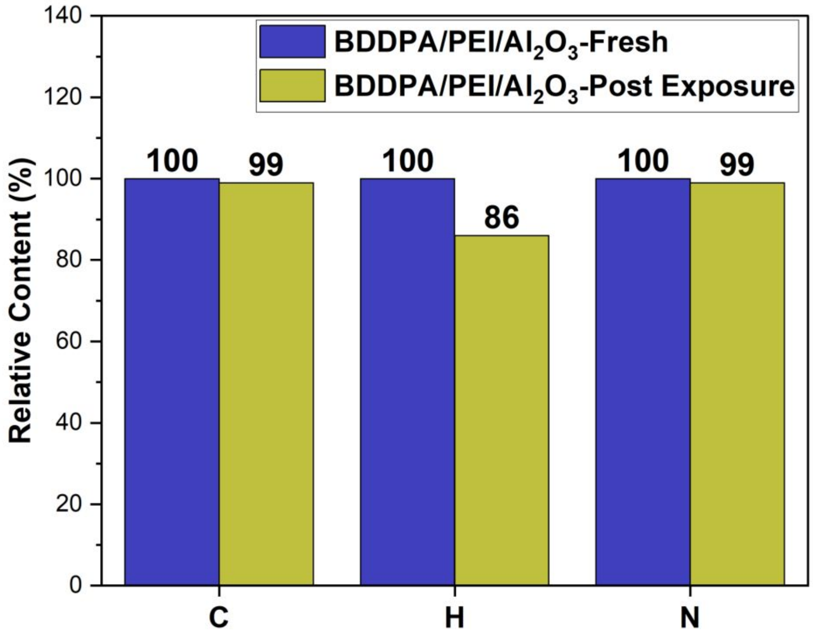

Figure S11. Changes in the carbon, hydrogen, and nitrogen content of the BDDPA/PEI/Al_2O_3 sorbent after 18 hours under humid 0.04% CO_2-air at 70 °C

Reference

(1) Gryn'ova, G.; Ingold, K. U.; Coote, M. L. New Insights into the Mechanism of Amine/Nitroxide Cycling during the Hindered Amine Light Stabilizer Inhibited Oxidative Degradation of Polymers. *J Am Chem Soc* **2012**, *134* (31), 12979-12988. DOI: 10.1021/ja3006379.
